# Shewanella phaeophyticola sp. nov. and Vibrio algarum sp. nov., isolated from marine brown algae

**DOI:** 10.1099/ijsem.0.006378

**Published:** 2024-05-10

**Authors:** Mahrukh Butt, Jaejoon Jung, Jeong Min Kim, Hülya Bayburt, Dong Min Han, Che Ok Jeon

**Affiliations:** 1 Department of Life Science, Chung-Ang University, Seoul 06974, Republic of Korea

**Keywords:** brown algae, new taxa, *Pseudomonadota*, *Shewanella phaeophyticola*, *Vibrio algarum*

## Abstract

Two Gram-stain-negative, rod-shaped bacteria, designated as strains KJ10-1^T^ and KJ40-1^T^, were isolated from marine brown algae. Both strains were catalase-positive, oxidase-positive, and facultative aerobic. Strain KJ10-1^T^ exhibited optimal growth at 25 °C, pH 7.0, and 3 % NaCl, whereas strain KJ40-1^T^ showed optimal growth at 25 °C, pH 7.0, and 2 % NaCl. The respiratory quinones of strain KJ10-1^T^ were ubiquinone-8, ubiquinone-7, menaquinone-7, and methylated menaquinone-7, while the respiratory quinone of strain KJ40-1^T^ was only ubiquinone-8. As major fatty acids, strain KJ10-1^T^ contained C_16 : 0_, C_17 : 1_ ω8*c*, iso-C_15 : 0_, and summed feature 3 (C_16 : 1_* ω*7*c* and/or C_16 : 1_* ω*6*c*) and strain KJ40-1^T^ contained C_16 : 0_ and summed features 3 and 8 (C_18 : 1_* ω*7*c* and/or C_18 : 1_* ω*6*c*). The major polar lipids in strain KJ10-1^T^ were phosphatidylethanolamine, phosphatidylglycerol, and an unidentified aminolipid, whereas those in strain KJ40-1^T^ were phosphatidylethanolamine, phosphatidylglycerol, and diphosphatidylglycerol. The DNA G+C contents of strains KJ10-1^T^ and KJ40-1^T^ were 42.1 and 40.8 mol%, respectively. Based on 16S rRNA gene sequences, strains KJ10-1^T^ and KJ40-1^T^ exhibited the closest relatedness to *Shewanella saliphila* MMS16-UL250^T^ (98.6 %) and *Vibrio rumoiensis* S-1^T^ (95.4 %), respectively. Phylogenetic analyses, based on both 16S rRNA and 92 housekeeping genes, showed that the strains formed distinct phylogenic lineages within the genera *Shewanella* and *Vibrio*. Digital DNA–DNA hybridization and orthologous average nucleotide identity values between strain KJ10-1^T^ and other *Shewanella* species, as well as between strain KJ40-1^T^ and other *Vibrio* species, were below the thresholds commonly accepted for prokaryotic species delineation. Based on the phenotypic, chemotaxonomic, and phylogenetic data, strains KJ10-1^T^ and KJ40-1^T^ represent novel species of the genera *Shewanella* and *Vibrio*, respectively, for which the names *Shewanella phaeophyticola* sp. nov. and *Vibrio algarum* sp. nov. are proposed, respectively. The type strains of *S. phaeophyticola* and *V. algarum* are KJ10-1^T^ (=KACC 22589^T^=JCM 35409^T^) and KJ40-1^T^ (=KACC 22588^T^=JCM 35410^T^), respectively.

## Introduction

The genus *Shewanella*, which belongs to the family *Shewanellaceae*, order *Alteromonadales*, phylum *Pseudomonadota*, was initially proposed by MacDonell and Colwell, with *Shewanella putrefaciens* as the type species [1]. As of March 2024, the genus encompasses a total of 108 species, both validly and invalidly published (https://lpsn.dsmz.de/genus/Shewanella), originating from diverse environments, including mangrove wetlands, fish, freshwater, seawater, marine sediments, marine algae, activated sludge, and tube worms [2,9]. Members of the genus *Shewanella* are typically Gram-stain-negative, oxidase- and catalase-positive, and facultative aerobic. Their cell morphology is characterized by straight or curved motile rods with a single, unsheathed, or polar flagellum. Additionally, these bacteria typically exhibit a non-pigmented to pale pink coloration. Additionally, they contain C_16 : 0_, C_17 : 1_ ω8*c*, iso-C_15 : 0_, and summed feature 3 (comprising C_16 : 1_* ω*7*c* and/or C_16 : 1_* ω*6*c*) as major fatty acids and ubiquinone-7 (Q-7), ubiquinone-8 (Q-8), menaquinone-7 (MK-7), and methylmenaquinone-7 (MMK-7) as respiratory quinones [2,9].

The genus *Vibrio*, with *Vibrio cholerae* as the type species, a member of the family *Vibrionaceae*, order *Vibrionales*, phylum *Pseudomonadota*, is one of the most diverse groups. As of March 2024, the genus encompasses a total of 179 species, both validly and invalidly published (https://lpsn.dsmz.de/genus/vibrio), originating from various environments, including marine and estuarine waters, as well as various organisms [10,17]. Members of the genus *Vibrio* are generally Gram-stain-negative, oxidase- and catalase-positive, facultative aerobic, and have straight or slightly curved cells, which may be motile or non-motile rods, with the capability to reduce nitrate to nitrite [10,17].

In this study, two putative novel bacterial strains, designated as strains KJ10-1^T^ and KJ40-1^T^, belonging to the genera *Shewanella* and *Vibrio*, respectively, were isolated from marine brown algae and subjected to taxonomic characterization using a polyphasic approach.

## Strain isolation

Strains KJ10-1^T^ and KJ40-1^T^ were isolated from brown algae *Ishige foliacea* and *Sargassum fusiforme*, respectively, which were collected from Gwangjin beach (37° 57′ 17.6″ N 128° 46′ 21.2″ E), Gangwon Province, Republic of Korea, in June 2021. The isolation process involved gentle washing of the collected brown algae with artificial seawater (ASW; 20 g NaCl, 2.9 g MgSO_4_, 4.53 g MgCl_2_·6H_2_O, 0.64 g KCl, and 1.75 g CaCl_2_·2H_2_O per litre). Subsequently, the washed algae were cut into 2–3 cm pieces, mechanically homogenized using an Ultra-Turrax Homogenizer (IKA) for 10 s in ASW, and serially diluted in ASW. Aliquots of 100 µl from each serial dilution were spread on marine agar (MA; MBcell), and the agar plates were incubated aerobically for 7 days at 25 °C. The 16S rRNA genes of colonies grown on MA were amplified by PCR using the universal primers F1 (5′-AGAGTTTGATCMTGGCTCAG-3′) and R13 (5′-TACGGYTACCTTGTTACGACTT-3′) [18]. The PCR amplicons were subjected to double digestion with *Hae*III and *Hha*I and subsequently analysed through 2 % agarose gel electrophoresis. PCR products displaying distinctive fragment patterns were partially sequenced using the universal primer 340F (5′-CCTACGGGAGGCAGCAG-3′) [18]. These sequences were compared with those of all type strains of validly published species on the EzBioCloud server (www.ezbiocloud.net/identify) [19].

Based on this analysis, two potential novel strains, designated as KJ10-1^T^ and KJ40-1^T^, were selected for further taxonomic characterization. Subsequently, strains KJ10-1^T^ and KJ40-1^T^ were purified by streaking on fresh MA three times. For a long-term storage, strains KJ10-1^T^ and KJ40-1^T^ were preserved at –80 °C in marine broth (MB; MBcell) supplemented with 15 % (v/v) glycerol and routinely cultured aerobically on MA or in MB for 2 days at 25 °C. To compare genomic characteristics, phenotypic properties, and fatty acid compositions, *Shewanella saliphila* JCM 32304^T^, *Shewanella algicola* JCM 31091^T^, *Shewanella inventionis* KCTC 42807^T^, *Vibrio hannami* KACC 19277^T^, *Vibrio rumoiensis* DSM 19141^T^, and *Vibrio marisflavi* DSM 23086^T^, obtained from their respective culture collection were used as reference strains.

## Phylogeny based on 16S rRNA gene sequences

The 16S rRNA gene amplicons from strains KJ10-1^T^ and KJ40-1^T^, generated using the F1 and R13 primers, were sequenced using the primers 518R (5′-ATTACCGCGGCTGCTGG-3′) and 805F (5′-GATTAGATACCCTGGTAGTC-3′) [18]. By assembling the sequences obtained using the 340F, 518R, and 805F primers, nearly complete 16S rRNA gene sequences were obtained for strains KJ10-1^T^ (1498 nucleotides) and KJ40-1^T^ (1493 nucleotides). Subsequently, the 16S rRNA gene sequence similarities between strains KJ10-1^T^ and KJ40-1^T^, as well as closely related type strains, were calculated using EzTaxon (www.ezbiocloud.net/identify) [19]. The alignment of the 16S rRNA gene sequences from strains KJ10-1^T^ and KJ40-1^T^ was conducted using the fast secondary-structure-aware infernal aligner (version 1.1.4) [20]. Phylogenetic trees, accompanied by bootstrap values derived from 1000 replications, were reconstructed using the neighbour-joining (NJ), maximum-likelihood (ML), and maximum-parsimony (MP) algorithms through the mega 11 software [21]. The Kimura two-parameter model, nearest-neighbour-interchange heuristic search method, and complete deletion options were used for the NJ, ML, and MP tree constructions, respectively.

**Fig. 1.**
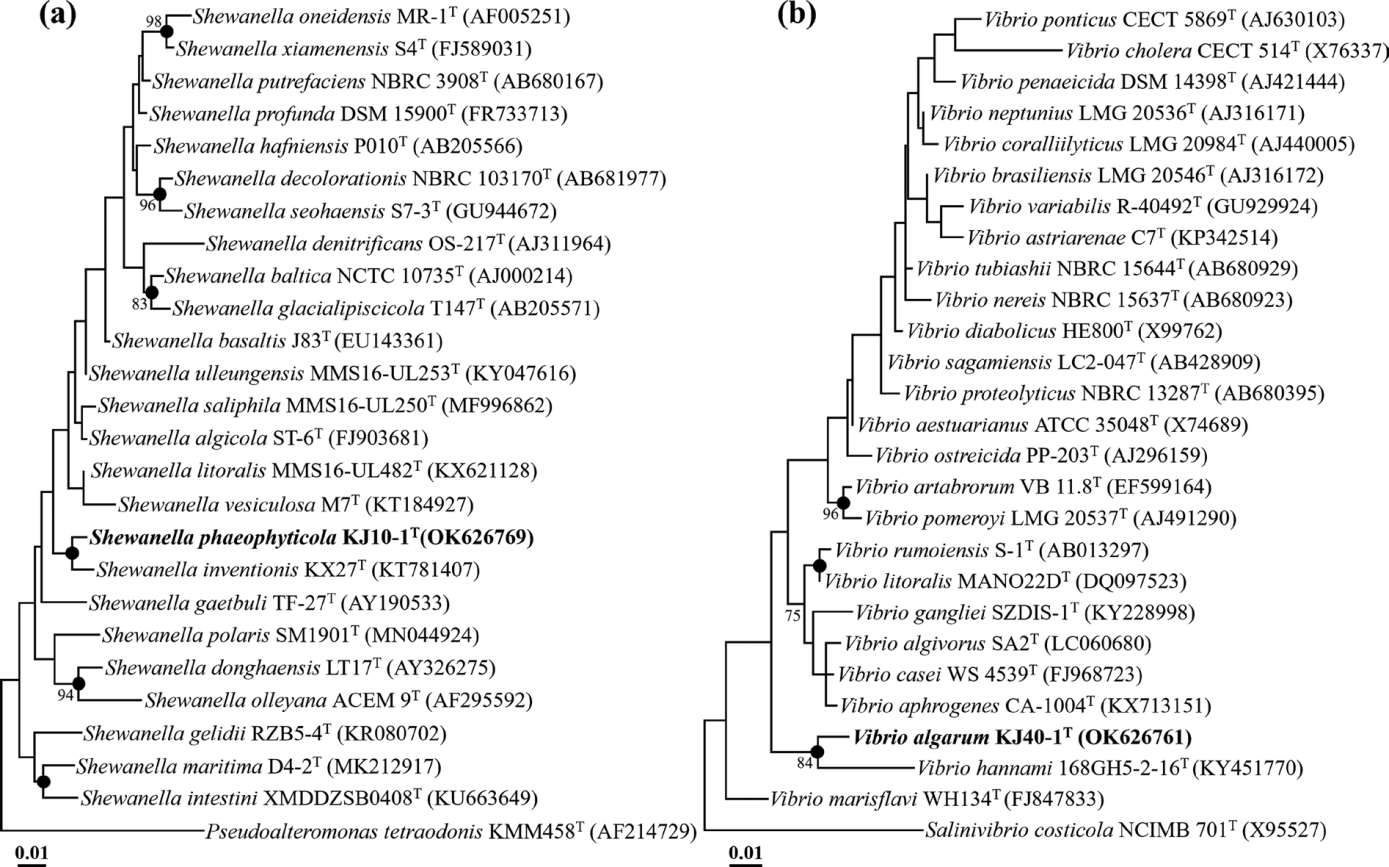
Maximum-likelihood trees based on 16S rRNA gene sequences, illustrating the phylogenetic relationships between strains KJ10-1^T^ (**a**) and KJ40-1^T^ (**b**) and their closely related taxa. The filled circles represent nodes that were also found in the neighbour-joining and maximum-parsimony trees. Bootstrap values greater than 70%, based on 1000 replicates, are indicated at the branching points. *Pseudoalteromonas tetraodonis* KMM 458^T^ (AF214729) and *Salinivibrio costicola* NCIMB 701^T^ (X95527) were used as the outgroups for the trees of strains KJ10-1^T^ and KJ40-1^T^, respectively. The scale bars indicate 0.01 changes per nucleotide position.

The comparative analysis of 16S rRNA gene sequences revealed that strain KJ10-1^T^ exhibited the highest sequence similarities of 98.6, 98.0, and 97.8 % to *S. saliphila* MMS16-UL250^T^, *S. inventionis* KX27^T^, and *S. algicola* ST-6^T^, respectively. On the other hand, strain KJ40-1^T^ showed the highest sequence similarities of 95.4, 94.8, and 94.5 % to *V. rumoiensis* S-1^T^, *V. hannami* 168GH5-2-16^T^, and *V. marisflavi* WH134^T^, respectively. In the phylogenetic tree reconstructed using the ML algorithm, it was observed that strain KJ10-1^T^ formed a phyletic lineage with *S. inventionis* KX27^T^ with a low bootstrap value within the genus *Shewanella*, while strain KJ40-1^T^ formed a phyletic lineage with *V. hannami* 168GH5-2-16^T^ with an 84 % bootstrap value within the genus *Vibrio* (Fig. 1). The phylogenetic trees, reconstructed using the NJ and MP algorithms, consistently indicated that strains KJ10-1^T^ and KJ40-1^T^ formed clusters with *S. inventionis* KX27^T^ and *V. hannami* 168GH5-2-16^T^ within their respective genera, exhibiting similar tree topologies (Fig. S1, available in the online version of this article). The combined results of comparative and phylogenetic analyses based on 16S rRNA gene sequences strongly suggest that strains KJ10-1^T^ and KJ40-1^T^ are indeed members of the genera *Shewanella* and *Vibrio*, respectively.

## Whole-genome sequencing and phylogenetic analysis based on 92 housekeeping genes

The genomic DNA of strains KJ10-1^T^ and KJ40-1^T^, as well as the reference strain *V. hannami* KACC 19277^T^, was extracted from cells cultured in MB using the Wizard Genomic DNA purification kit from Promega, following the manufacturer’s instructions. The genomic DNA was subsequently sequenced on an Oxford Nanopore MinION sequencer. The obtained sequencing reads were subjected to *de novo* assembly using Flye (version 2.9.1) [22], and the quality of the assembled genomes was assessed based on their completeness and contamination rates using the CheckM program (version 1.0.4) [23].

For the phylogenomic analysis of strains KJ10-1^T^ and KJ40-1^T^, a set of 92 pre-defined single-copy housekeeping core genes was extracted from their genomes, as well as from their closely related type strains, using the bacterial core gene pipeline available at www.ezbiocloud.net/tools/ubcg [24]. Subsequently, a phylogenomic ML tree with bootstrap values (1000 replications) was reconstructed based on the concatenated amino acid sequences of these core genes using the mega11 software. Additionally, the orthologous average nucleotide identity (OrthoANI) and digital DNA–DNA hybridization (dDDH) values were calculated between strain KJ10-1^T^ and its closely related *Shewanella* type strains, as well as between strain KJ40-1^T^ with its closely related *Vibrio* type strains. The OrthoANI calculations were performed using the Orthologous Average Nucleotide Identity Tool software available on the EzBioCloud server (www.ezbiocloud.net/sw/oat) [25]. The dDDH calculations were performed using the Genome-to-Genome Distance Calculator version 2.1 (https://ggdc.dsmz.de/distcalc2.php) [26].

The genomes of strains KJ10-1^T^, KJ40-1^T^, and KACC 19277^T^ were sequenced with average genome coverages of 46.6×, 396.0×, and 14.0×, respectively. The *de novo* assembly of the genome sequencing data for strains KJ10-1^T^, KJ40-1^T^, and KACC 19277^T^ resulted in draft genomes sizes of 4.8, 4.9, and 5.4 Mb composed of three, three, and 10 contigs, respectively; the N50 values for these genomes were 4763, 3253, and 1999 kb. The 16S rRNA gene sequences in the genomes of strains KJ10-1^T^, KJ40-1^T^, and KACC 19277^T^ were identical to those obtained through PCR-based sequencing. The completeness and contamination rates of the assembled genomes for strains KJ10-1^T^, KJ40-1^T^, and KACC 19277^T^ were determined to be 96.5, 95.2, and 97.8 % for completeness, and 0.3, 4.6, and 5.2 % for contamination, respectively, which met the criteria representing generally high-quality genomes (90 % or higher completeness; 10 % or lower contamination) [27]. The draft genome sequences for strains KJ10-1^T^, KJ40-1^T^, and KACC 19277^T^ have been deposited in GenBank under the accession numbers JAODOQ000000000, JAQLOI000000000, and JARQZP000000000, respectively.

**Fig. 2.**
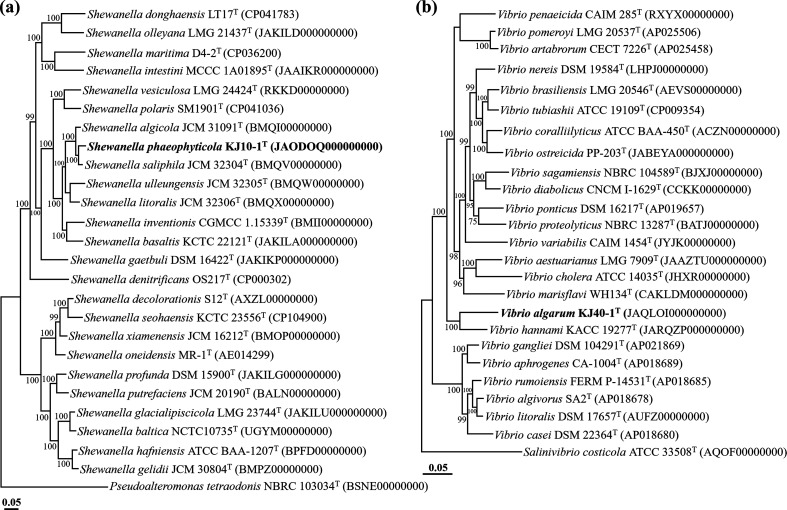
Maximum-likelihood phylogenomic trees based on the concatenated amino acid sequences of 92 housekeeping core genes, illustrating the phylogenetic relationships between strains KJ10-1^T^ (**a**) and KJ40-1^T^ (**b**) and their closely related taxa. Bootstrap values greater than 70%, based on 1000 replicates, are shown at the branching points. *Pseudoalteromonas tetraodonis* NBRC 103034^T^ (BSNE00000000) and *Salinivibrio costicola* ATCC 33508^T^ (AQOF00000000) were used as outgroups for the trees of strains KJ10-1^T^ and KJ40-1^T^, respectively. The scale bars indicate 0.05 changes per amino acid.

The phylogenomic analysis based on 92 housekeeping genes demonstrated that strains KJ10-1^T^ and KJ40-1^T^ formed robust phylogenetic lineages with *S. saliphila* JCM 32304^T^ and *V. hannami* KACC 19277^T^, respectively, within the genera *Shewanella* and *Vibrio*, with both lineages showing 100 % bootstrap values (Fig. 2). This further confirms the conclusion drawn from the analyses based on 16S rRNA gene sequences, affirming that strains KJ10-1^T^ and KJ40-1^T^ indeed belong to the genera *Shewanella* and *Vibrio*, respectively. The OrthoANI and dDDH values between strain KJ10-1^T^ and its closely related type strains *S. saliphila* JCM 32304^T^, *S. algicola* JCM 31091^T^, and *S. inventionis* CGMCC 1.15339^T^ were 92.4, 91.2, and 84.8 % for OrthoANI, and 47.4, 43.4, and 28.8 % for dDDH, respectively (Table S1). Similarly, the OrthoANI and dDDH values between strain KJ40-1^T^ and its closely related type strains *V. hannami* KACC 19277^T^, *V. rumoiensis* FERM P-14531^T^, and *V. marisflavi* WH134^T^ were 71.9, 70.9, and 70.5 % for OrthoANI, and 20.8, 24.1, and 23.1 % for dDDH, respectively (Table 1). These values fall below the established thresholds (OrthoANI, ~95 %; dDDH, 70 %) for prokaryotic species delineation [27]. The results from the phylogenomic analysis and genome relatedness assessments strongly support the conclusion that strains KJ10-1^T^ and KJ40-1^T^ represent novel species within the genera *Shewanella* and *Vibrio*, respectively.

**Table 1.** General genomic features* of strains KJ10-1^T^ and KJ40-1^T^, as well as closely related type strains of the genera *Shewanella* and *Vibrio* Strains: 1, KJ10-1^T^ (JAODOQ000000000); 2, *S. saliphila* JCM 32304^T^ (BMQV00000000); 3, *S. algicola* JCM 31091^T^ (BMQI00000000); 4, *S. inventionis* CGMCC 1.15339^T^ (BMII00000000); 5, KJ40-1^T^ (JAQLOI000000000); 6, *V. hannami* KACC 19277^T^ (JARQZP000000000); 7, *V. rumoiensis* FERM P-14531^T^ (AP018685); 8, *V. marisflavi* WH134^T^ (CAKLDM000000000). The genomes of strains KJ10-1^T^ and KJ40-1^T^ and *V. hannami* KACC 19277^T^ were sequenced in this study. –, Not calculated.

Features	1	2	3	4	5	6	7	8
Genome size (Mb)	4.8	4.6	4.9	5.0	4.9	5.4	4.2	4.8
G+C contents (mol%)	42.1	42.5	42.3	42.1	40.8	43.8	42.3	42.0
N50 (kb)	4763	81	80	124	3253	1999	2763	2906
No. of contigs	3	126	175	141	3	10	4	8
No. of total genes	4464	4093	4470	4417	4552	5123	3845	4436
No. of protein-coding genes	3466	3938	4282	4211	4113	4670	3628	4250
No. of tRNA genes	99	85	84	88	95	95	91	105
No. of rRNA genes	25	6	1	8	28	29	25	33
No. of ncRNA genes	3	3	3	3	3	3	3	3
No. of tmRNA genes	1	1	1	1	1	1	1	1
No. of total CAZyme† genes:	68	55	55	73	88	103	65	69
Glycoside hydrolases	26	24	26	28	39	58	27	32
Glycosyltransferases	18	14	13	21	28	29	19	23
Polysaccharide lyases	6	6	6	7	7	6	8	0
Carbohydrate esterases	12	9	6	11	9	8	5	7
Auxiliary activities	3	1	3	3	2	1	5	4
Carbohydrate-binding modules	3	1	1	3	3	1	1	4

*The genomic features except for CAZyme information were obtained from the NCBI nucleotide (www.ncbi.nlm.nih.gov/nucleotide/) and assembly (www.ncbi.nlm.nih.gov/assembly) databases.

†CAZyme, Ccarbohydrate-active enzyme.

## Genomic features

The genomes of strains KJ10-1^T^ and KJ40-1^T^, as well as the reference strains, were annotated using the NCBI Prokaryotic Genome Annotation Pipeline [28]. Additionally, the carbohydrate-active enzymes (CAZymes) were analysed using the dbCAN2 meta server [29]. Strain KJ10-1^T^ was predicted to have a total of 4464 genes, while strain KJ40-1^T^ had 4552 genes. Among these, 3466 and 4113 were protein-coding genes, 99 and 95 were tRNA genes, 25 and 28 were rRNA genes, and three each were non-coding RNA genes, respectively (Table 1). The DNA G+C contents, calculated based on the entirety of their respective genomes, were 42.1 mol% for strain KJ10-1^T^ and 40.8 mol% for strain KJ40-1^T^. These values fall within the range of G+C contents observed in their closely related type strains (Table 1). A comprehensive summary and comparison of the general genomic features of strains KJ10-1^T^ and KJ40-1^T^ with those of closely related type strains from the genera *Shewanella* and *Vibrio* can be found in Table 1.

Bacteria inhabiting the phycosphere of marine algae may possess the capability to metabolize cell-wall components from the algae to support their growth. Some species of *Shewanella* and *Vibrio* have been previously reported to exhibit the ability to degrade various components of algal cell walls [30,31]. Therefore, we conducted a genome-wide analysis to examine the distribution of genes encoding CAZymes, which are potentially associated with the breakdown of algal cell-wall components, in the genomes of strains KJ10-1^T^ and KJ40-1^T^, as well as their respective reference strains. This analysis revealed a total of 68 putative CAZyme-encoding genes in the genome of strain KJ10-1^T^, while strain KJ40-1^T^ had 88 such genes. These numbers generally exceeded those observed in the reference strains of *Shewanella* and *Vibrio* (Table 1). This suggests that strains KJ10-1^T^ and KJ40-1^T^ isolated from marine algae may possess enhanced capabilities to utilize cell-wall components from marine algae compared to other species of *Shewanella* and *Vibrio*. Notably, genes belonging to the glycoside hydrolase (GH) and glycosyltransferase (GT) families were abundantly identified among the six major CAZyme categories. Strain KJ10-1^T^ and its reference strains commonly had genes from the GH103, GH23, and GT51 families, which encode peptidoglycan lyase, peptidoglycan transglycosylase, and peptidoglycan glycosyltransferase, respectively. However, only strain KJ10-1^T^ had the polysaccharide lyase (PL) 17 family gene, which corresponds to alginate lyase, suggesting its differential ability to decompose alginate. On the other hand, strain KJ40-1^T^ and its reference strains commonly possess GH23 family genes corresponding to lysozyme, peptidoglycan lyase, and chitinase. However, strain KJ40-1^T^ did not have the GH1 family gene, corresponding to *β*-glycoside hydrolases, unlike other *Vibrio* reference strains, suggesting its limited ability to decompose glycan compared to its reference strains.

## Morphology and phenotypic properties

The growth ability of strains KJ10-1^T^ and KJ40-1^T^ on various standard bacteriological agar media (all sourced from MBcell), including MA, Reasoner’s 2A (R2A) agar, Luria–Bertani (LB) agar, tryptic soy agar (TSA), and nutrient agar (NA), was evaluated. The NaCl concentrations in R2A agar, LB agar, TSA, and NA were adjusted to 2 % (w/v). To assess growth temperature and pH, the strains were cultivated at different temperatures (4 °C, 10–40 °C at 5 °C intervals) and pH values (ranging from pH 4.0 to 11.0 in 1.0 pH intervals) on MA and in MB, respectively. For pH adjustment, sodium citrate, Na_2_HPO_4_/NaH_2_PO_4_, Tris-HCl, and NaOH buffer systems were used to prepare MB media with pH values of 4.0–5.0, 6.0–7.0, 8.0–9.0, and 10.0–11.0, respectively [32]. The pH levels were adjusted after autoclaving (at 121 °C for 15 min). Salt tolerance was assessed in MB with varying NaCl concentrations (ranging from 0–10 % at 1.0 % intervals, w/v) prepared in the laboratory according to the MB formula: 5 g peptone, 1 g yeast extract, 0.1 g ferric citrate, 5.9 g MgCl_2_·6H_2_O, 3.24 g Na_2_SO_4_, 1.8 g CaCl_2_·2H_2_O, 0.55 g KCl, 0.16 g NaHCO_3_, 80 mg KBr, 34 mg SrCl_2_·6H_2_O, 22 mg H_3_BO_3_, 4 mg Na_2_SiO_3_·5H_2_O, 2.4 mg NaF, 1.6 mg NH_4_NO_3_, and 8 mg Na_2_HPO_4_·12H_2_O, with variable NaCl concentration per litre. Physiological and biochemical tests for strains KJ10-1^T^ and KJ40-1^T^ were conducted using cells that were incubated at 25 °C for 2 days. Cell morphology, size, and the presence of flagella were examined through transmission electron microscopy (JEM-1010, jeol) and phase-contrast microscopy (Axio Scope.A1, Carl Zeiss). Gram staining was performed using a Gram stain kit from bioMérieux, following the manufacturer’s instructions. Anaerobic growth was assessed on MA for a duration of 21 days at 25 °C under anaerobic conditions created using the GasPak Plus system (BBL). Oxidase activity was evaluated by observing the oxidation of 1 % (w/v) tetramethyl-*p*-phenylenediamine (Merck), while catalase activity was determined by the production of oxygen bubbles in a 3 % (v/v) aqueous hydrogen peroxide solution. The phenotypic characteristics of strains KJ10-1^T^ and KJ40-1^T^ were examined alongside reference strains under the same conditions at their respective optimal temperatures. Hydrolysis of casein (1 % skimmed milk, w/v), starch (1 %), aesculin (0.1 %), l-tyrosine (0.5 %), Tween 20 (1 %), and Tween 80 (1 %) was assessed on MA, following previously established protocols [33]. Additional biochemical features and enzymatic activities were evaluated using the API 20NE and API ZYM kits from bioMérieux, following the manufacturer’s instructions, except that the solutions in the API kits were adjusted to approximately 2 % NaCl.

Strain KJ10-1^T^ exhibited optimal growth on MA, and also demonstrated relatively good growth on R2A agar, TSA, and NA with 2 % NaCl. However, it exhibited poor growth on LB agar with 2 % NaCl. In contrast, strain KJ40-1^T^ exhibited optimal growth on MA, relatively good growth on TSA and NA with 2 % NaCl, but poor growth on R2A agar and LB agar with 2 % NaCl. Morphologically, cells of strain KJ10-1^T^ were characterized as motile rods with a single polar flagellum, measuring 1.0–1.3×2.0–2.7 µm, whereas cells of strain KJ40-1^T^ were non-motile rods, measuring 0.5–0.6×1.2–2.0 µm in size (Fig. S2). Both strains displayed minor anaerobic growth, suggesting their facultative aerobic nature. While strains KJ10-1^T^ and KJ40-1^T^, along with their closely related respective reference strains, shared several phenotypic, physiological, and biochemical features (e.g., flagellum motility, oxidase and catalase activities, nitrate reduction, and indole production for strain KJ10-1^T^; d-glucose fermentation, oxidase and catalase activities, and hydrolysis of aesculin and l-tyrosine for strain KJ40-1^T^), there were also notable differences (e.g., colony colour, growth conditions, and hydrolysis of starch, casein, l-tyrosine, and Tween 20 for strain KJ10-1^T^; colony colour, growth conditions, flagellum motility, nitrate reduction, and indole production for strain KJ40-1^T^). These differences enable the differentiation of strains KJ10-1^T^ and KJ40-1^T^ from their respective reference strains (Tables2  3).

**Table 2.** Differential phenotypic characteristics observed between strain KJ10-1^T^ and closely related type strains of the genus *Shewanella* Strains: 1, KJ10-1^T^ (this study); 2, *S. saliphila* JCM 32304^T^ [2]; 3, *S. algicola* JCM 31091^T^ [4]; 4, *S. inventionis* KCTC 42807^T^ [3]. All strains were positive for the following characteristics: nitrate reduction, flagellum motility*, activity of oxidase, catalase, alkaline phosphatase, esterase lipase (C8), leucine arylamidase, valine arylamidase, and *α*-chymotrypsin, hydrolysis of aesculin, assimilation of d-mannitol and maltose. All strains were negative for the following characteristics: indole production, d-glucose fermentation, activity of urease, arginine dihydrolase, *β*-galactosidase, *β-*glucuronidase, *β*-glucosidase, *α*-mannosidase, *N*-acetyl-*β*-glucosaminidase, and *α*-fucosidase, assimilation of l-arabinose, d-mannose, *N*-acetyl-glucosamine, potassium gluconate, capric acid, adipic acid, trisodium citrate, and phenylacetic acid. +, Positive; –, negative; w, weakly positive.

Characteristic	1	2	3	4
Isolation source*	Brown alga	Seawater	Brown alga	Seawater
Colony colour*	Orange	Pale pink	Pale pink	Pale pink
Growth range (optimum):*				
Temperature (°C)	4–30 (25)	4–30 (25)	4–30 (20)	4–30 (25)
NaCl (%)	2–8 (3)	2–8 (4.5)	1–6 (2)	1–6 (3)
pH	6–8 (7)	6–10 (7)	6–10 (7.5)	6–9 (7–8)
Hydrolysis of:				
Starch	–	+	–	+
Casein, l-tyrosine, Tween 20, Tween 80	+	+	+	–
Assimilation of:				
d-Glucose	+	–	+	–
Malic acid	–	+	+	+
Enzyme activity of:				
Esterase (C4), cystine arylamidase, trypsin, naphthol-AS-BI-phosphohydrolase	+	+	+	–
Lipase (C14), gelatinase	–	–	+	–
*α*-Galactosidase	–	–	+	+
Acid phosphatase	+	w	–	+
*α*-Glucosidase	*+*	–	+	+

*Data marked with an asterisk were obtained from previous studies for the respective strains.

**Table 3.** Differential phenotypic characteristics observed between strain KJ40-1^T^ and closely related type strains of the genus *Vibrio* Strains: 1, KJ40-1^T^ (this study); 2, *V. hannami* KACC 19277 ^T^ [6]; 3, *V. rumoiensis* DSM 19141^T^ [7]; 4, *V. marisflavi* DSM 23086^T^ [8]. All strains were positive for the following characteristics: d-glucose fermentation, activity of oxidase, catalase, alkaline phosphatase, esterase (C4), esterase lipase (C8), acid phosphatase, naphthol-AS-BI-phosphohydrolase, *α-*glucosidase, and *β*-glucosidase, and hydrolysis of aesculin. All strains were negative for the following characteristics: enzyme activity of trypsin, *α*-chymotrypsin, *N*-acetyl-*β*-glucosaminidase, and *α*-galactosidase, hydrolysis of l-tyrosine, and assimilation of capric acid, adipic acid, trisodium citrate, and phenylacetic acid. +, Positive; –, negative.

Characteristic	1	2	3	4
Isolation source*	Brown alga	Seawater	Seaweed	Seawater
Colony colour*	Light-yellow	Non-pigmented	White	Light-yellow
Flagellum motility*	–	+	–	+
Nitrate reduction to nitrite	+	–	+	+
Indole production	+	+	–	–
Growth range (optimum):*				
Temperature (°C)	10–30 (25)	20–42 (25)	20–37 (25)	16–37 (30)
NaCl (%)	1–8 (2)	2–8 (2)	1–6 (3)	1–6 (2)
pH	6–10 (7)	6–10 (6)	6–10 (7.5)	6–10 (7.5–8.5)
Hydrolysis of:				
Starch, Tween 20, Tween 80	–	–	+	+
Casein	–	–	–	+
Assimilation of:				
Maltose, *N*-acetyl-glucosamine	–	+	–	+
d-Mannose	–	–	–	+
Malic acid	+	*+*	–	–
Potassium gluconate, d-mannitol	–	–	+	+
d-Glucose, l-arabinose	–	*+*	+	+
Enzyme activity of:				
Lipase (C14)	+	–	–	+
Arginine dihydrolase	–	+	–	+
Urease, gelatinase	–	–	–	+
Leucine arylamidase	–	+	–	–
Valine arylamidase, cystine arylamidase, *β*-glucuronidase	+	–	–	–
*α*-Mannosidase, *α*-fucosidase	–	–	+	–
*β*-Galactosidase	–	+	+	–

*Data marked with an asterisk were obtained from previous studies for the respective strains.

## Chemotaxonomy

The respiratory isoprenoid quinones from strains KJ10-1^T^ and KJ40-1^T^ were extracted according to the method described by Minnikin *et al*. [34] and analysed using an HPLC system (LC-20A, Shimadzu) equipped with a reversed-phase column (250×4.6 mm; Kromasil, Akzo Nobel) and a diode array detector (SPD-M20A, Shimadzu). Methanol–isopropanol (2 : 1, v/v) was used as the eluent and the flow rate was 1 ml min^−1^.

For the analysis of cellular fatty acids, the cells of strains KJ10-1^T^ and KJ40-1^T^, along with their reference strains, were aerobically cultivated in MB at their respective optimal temperatures and harvested during their exponential growth phases (when optical density reached 0.8 at 600 nm). The cellular fatty acids were subsequently extracted from the harvested cells, saponified, and methylated following the standard midi protocol. The resulting fatty acid methyl esters were analysed using a 6890-gas chromatograph (Hewlett Packard) and identified using the RTSBA6 database of the Microbial Identification System (Sherlock version 6.0B) [35].

The polar lipids of strains KJ10-1^T^ and KJ40-1^T^ were extracted from cells harvested during their exponential growth phases and analysed according to the procedure outlined by Minnikin *et al*. [36], using a two-dimensional thin-layer chromatography method. The following reagents were used to identify different types of polar lipids: 10 % ethanolic molybdophosphoric acid (for total polar lipids), ninhydrin (for aminolipids), Dittmer–Lester reagent (for phospholipids), and *α*-naphthol/sulphuric acid (for glycolipids). The presence of phosphatidylethanolamine (PE), phosphatidylglycerol (PG), and diphosphatidylglycerol (DPG) in strains KJ10-1^T^ and KJ40-1^T^ was confirmed using standard polar lipid compounds purchased from Sigma-Aldrich.

The respiratory quinones Q-8 (44.0 %), Q-7 (36.2 %), MK-7 (11.3 %), and MMK-7 (8.5 %) were detected in strain KJ10-1^T^. The presence of ubiquinone as the major quinone and menaquinone as the minor quinone aligns with the characteristic quinone pattern observed in various members of the genus *Shewanella* [2,5, 7,9]. Nonetheless, the specific proportions of these quinones vary among different *Shewanella* species [2,5]. On the other hand, the only respiratory quinone detected in strain KJ40-1^T^ was Q-8, consistent with previous reports on *Vibrio* species [16,18].

In strain KJ10-1^T^, the major cellular fatty acids (at least 10 % each) were C_16 : 0_, C_17 : 1_* ω*8*c*, iso-C_15 : 0_, and summed feature 3 (comprising C_16 : 1_* ω*7*c* and/or C_16 : 1_* ω*6*c*; Table S1); whereas in strain KJ40-1^T^, the major cellular fatty acids were C_16 : 0_ and summed features 3 and 8 (comprising C_18 : 1_* ω*7*c* and/or C_18 : 1_* ω*6*c*; Table S2). While the overall fatty acid profiles of strains KJ10-1^T^ and KJ40-1^T^ were generally similar to those of their closely related reference *Shewanella* and *Vibrio* strains, there were some differences in the proportions of certain fatty acids (Tables S2 and S3). For instance, C_16 : 0_ and C_17 : 1_* ω*8*c* were predominantly found in strain KJ10-1^T^, whereas they were detected only in trace amounts in *S. saliphila* JCM 32304^T^. Additionally, C_12 : 0_ 3-OH was present in trace amounts in strain KJ10-1^T^, but in other reference *Shewanella* strains, it was present in amounts exceeding 2 %. On the other hand, iso-C_15 : 0_, C_18 : 1_ *ω*9*c* and C_18 : 1_* ω*7*c* were not detected in strain KJ40-1^T^, but they were identified in *V. hannami* KACC 19277^T^, *V. rumoiensis* DSM 19141^T^, and *V. marisflavi* DSM 23086^T^, although the detected amounts differed significantly among them.

The major polar lipids identified in strain KJ10-1^T^ were PE, PG, and an unidentified aminolipid, while those in strain KJ40-1^T^ were PE, PG, and DPG (Fig. S3). These results are consistent with previous reports on closely related species of *Shewanella* [7,9] and *Vibrio* [16,18], which also found PE and PG to be the main polar lipids in strain KJ10-1^T^, and PE, PG, and DPG to be dominant in strain KJ40-1^T^.

## Taxonomic conclusion

The phylogenetic inference, as well as the phenotypic and chemotaxonomic characteristics, support the notion that strains KJ10-1^T^ and KJ40-1^T^ represent novel species within the genera *Shewanella* and *Vibrio*, respectively. Therefore, we propose the species names *Shewanella phaeophyticola* sp. nov. and *Vibrio algarum* sp. nov.

## Description of *Shewanella phaeophyticola* sp. nov.

*Shewanella phaeophyticola* [phae.o.phy.ti′co.la. N.L. neut. pl. n. *Phaeophyta* the division of the brown algae; L. suff. –*cola* (from L. n. *incola*) inhabitant, dweller; N.L. fem. n. *phaeophyticola*, inhabitant of *Phaeophyta*].

Colonies on MA are orange-coloured, circular, smooth, shiny, and convex with a diameter of 0.5–1.0 mm after 2 days of incubation at 25 °C. Cells are Gram-stain-negative, facultative aerobic, and motile rods with a single polar flagellum. Growth occurs at 4–30 °C (optimum, 25 °C) and pH 6.0–8.0 (optimum, pH 7.0) and in the presence of 2.0–8.0% NaCl (optimum, 3.0 %). Oxidase-positive and catalase-positive. Nitrate is reduced to nitrite. Aesculin, casein, l-tyrosine, Tween 20, and Tween 80 are hydrolysed, but starch is not. Indole production and d-glucose fermentation are negative. Activity of alkaline phosphatase, esterase (C4), esterase lipase (C8), leucine arylamidase, valine arylamidase, cystine arylamidase, trypsin, *α*-chymotrypsin, acid phosphatase, naphthol-AS-BI-phosphohydrolase, and *α*-glucosidase is positive, but activity of gelatinase, arginine dihydrolase, urease, lipase (C14), *α*-galactosidase, *β*-glucuronidase, *β*-glucosidase, *β*-galactosidase, *N*-acetyl-*β*-glucosaminidase, *α*-mannosidase, and *α*-fucosidase is negative. Assimilation of d-mannitol, d-glucose, and maltose is positive, but assimilation of l-arabinose, d-mannose, *N*-acetyl-glucosamine, potassium gluconate, adipic acid, capric acid, malic acid, trisodium citrate, and phenylacetic acid is negative. Q-8, Q-7, MK-7, and MMK-7 are identified as respiratory quinones. The major fatty acids are C_16 : 0_, C_17 : 1_* ω*8*c*, iso-C_15 : 0_, and summed feature 3 (C_16 : 1_* ω*7*c* and/or C_16 : 1_* ω*6*c*). PE, PG, and AL are identified as the major polar lipids.

The type strain is KJ10-1^T^ (=KACC 22589^T^=JCM 35409^T^), isolated from a brown alga *Ishige foliacea* collected in the Republic of Korea. The genome size and DNA G+C content of the type strain are 4.8 Mb and 42.1 mol% (calculated from the whole genome sequence), respectively. The GenBank accession numbers for the 16S rRNA gene and genome sequences of strain KJ10-1^T^ are OK626769 and JAODOQ000000000, respectively.

## Description of *Vibrio algarum* sp. nov.

*Vibrio algarum* (al.ga′rum. L. gen. pl. n. *algarum*, of/from algae).

Colonies on MA are smooth, circular, light yellow, and slightly convex with a diameter of 0.5–1.0 mm after 2 days of incubation at 25 °C. Cells are Gram-stain-negative, facultative aerobic, and non-motile rods. Growth occurs at 10–30 °C (optimum, 25 °C) and pH 6.0–10.0 (optimum, pH 7.0) and in the presence of 1.0–8.0% NaCl (optimum, 2.0 % NaCl). Oxidase-positive and catalase-positive. Nitrate is reduced to nitrite. Indole is produced and d-glucose is fermented. Aesculin is hydrolysed, but l-tyrosine, casein, starch, Tween 20, and Tween 80 are not. Activity of alkaline phosphatase, esterase (C4), esterase lipase (C8), lipase (C14), valine arylamidase, cystine arylamidase, acid phosphatase, naphthol-AS-BI-phosphohydrolase, *β*-galactosidase, *α*-glucosidase, and *β*-glucosidase is positive, but activity of gelatinase, arginine dihydrolase, urease, leucine arylamidase, trypsin, *α*-chymotrypsin, *α-*galactosidase, *β*-glucuronidase, *N*-acetyl-*β*-glucosaminidase, *α*-mannosidase, and *α*-fucosidase is negative. Assimilation of malic acid is positive, but assimilation of d-glucose, l-arabinose, d-mannose, d-mannitol, *N*-acetyl-glucosamine, maltose, potassium gluconate, adipic acid, capric acid, trisodium citrate, and phenylacetic acid is negative. The major fatty acids are C_16 : 0_, summed feature 3 (C_16 : 1_* ω*7*c* and/or C_16 : 1_* ω*6*c*), and summed feature 8 (C_18 : 1_* ω*7*c* and/or C_18 : 1_* ω*6*c*). Q-8 is the sole respiratory quinone. PE, PG, and DPG are identified as the major polar lipids.

The type strain is KJ40-1^T^ (=KACC 22588^T^=JCM 35410^T^), isolated from a brown alga *Sargassum fusiforme* collected in the Republic of Korea. The genome size and DNA G+C content of the type strain are 4.9 Mb and 40.8 mol% (calculated from the whole genome sequence), respectively. The GenBank accession numbers for the 16S rRNA gene and genome sequences of strain KJ40-1^T^ are OK626761 and JAQLOI000000000, respectively.

## supplementary material

10.1099/ijsem.0.006378 Uncited Supplementary Material 1.

## Abbreviations

dDDH: digital DNA–DNA hybridization
DPG: diphosphatidylglycerol
LB: Luria–Bertani
MA: marine agar
MA: marine agar
MB: marine broth
MK-7: menaquinone-7
ML: maximum-likelihood
MMK-7: methylated menaquinone-7
MP: maximum-parsimony
NA: nutrient agar
NJ: neighbour-joining
OrthoANI: orthologous average nucleotide identity
PE: phosphatidylethanolamine
PG: phosphatidylglycerol
Q-7: ubiquinone-7
Q-8: ubiquinone-8
R2A: Reasoner’s 2A
TSA: tryptic soy agar
