# Supplementary material for: Shewanella phaeophyticola sp. nov. and Vibrio algarum sp. nov., isolated from marine brown algae
Source: Int J Syst Evol Microbiol. 2024 May 10;74(5):006378. doi: 10.1099/ijsem.0.006378 (PMC11165915; doi:10.1099/ijsem.0.006378)
Supplement: Uncited Supplementary Material 1. [file ijsem-74-06378-s001.pdf]

Supplementary Information

Fig. S1. Neighbor-joining (NJ; a and b) and maximum parsimony (MP; c and d) trees based on 16S rRNA gene sequences showing the phylogenetic relationships between strains KJ10-1^{T} (a and c) and KJ40-1^{T} (b and d) and their closely related taxa. Bootstrap values (>70%) based on 1000 replicates are shown on branch nodes. *Pseudoalteromonas tetraodonis* KMM458^{T} (AF214729) and *Salinivibrio costicola* NCIMB 701^{T} (X95527) were used as the outgroups for the genera *Shewanella* and *Vibrio*, respectively. The scale bars in the NJ and MP trees indicate nucleotide changes per nucleotide position and nucleotide changes over the whole 16S rRNA sequences, respectively.

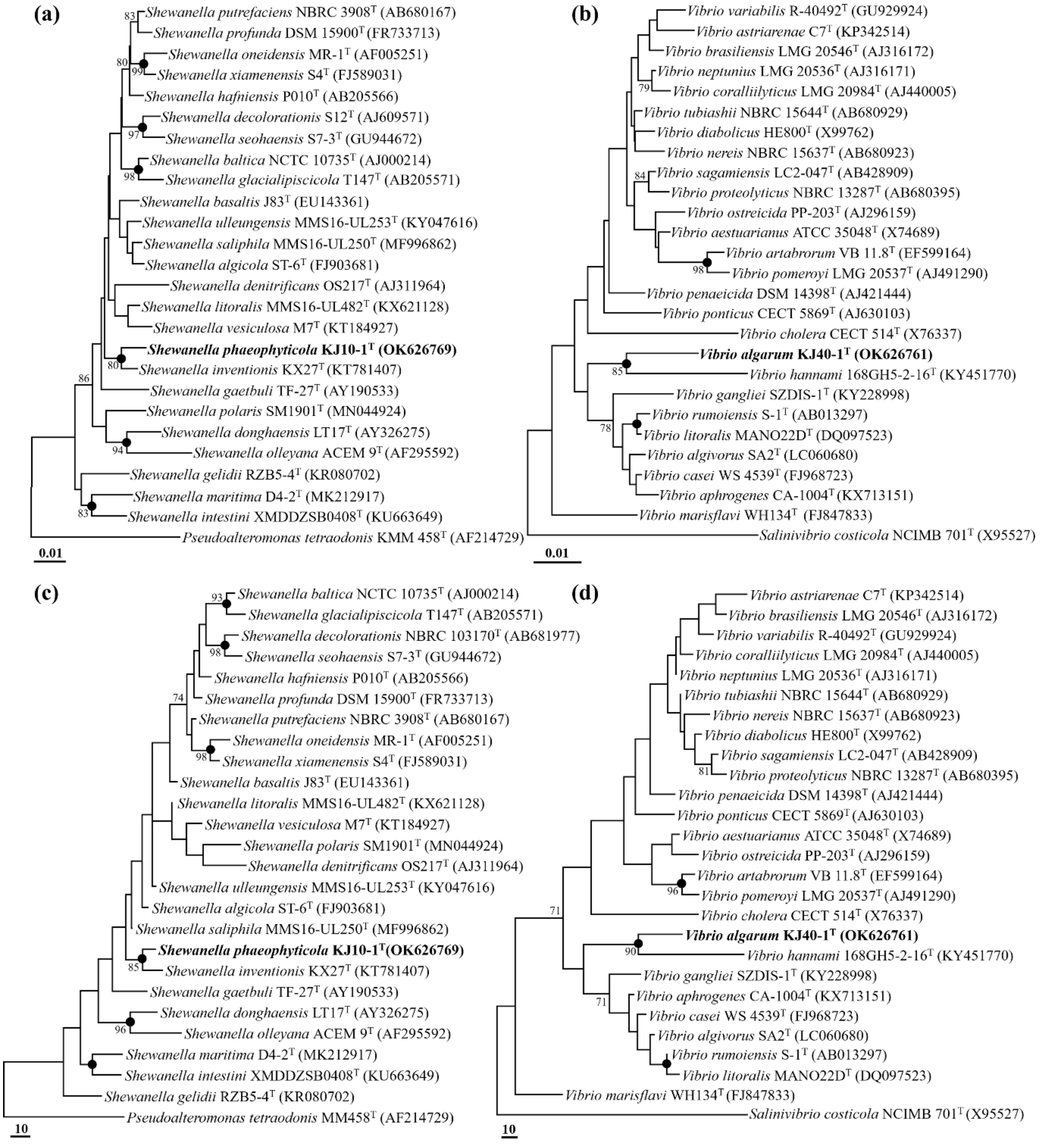

Fig. S2. Transmission electron micrographs of negatively stained (using 2% uranyl acetate) cells showing the general morphologies of strains KJ10-1^T (a) and KJ40-1^T (b) grown on marine agar at 25°C for 2 days.

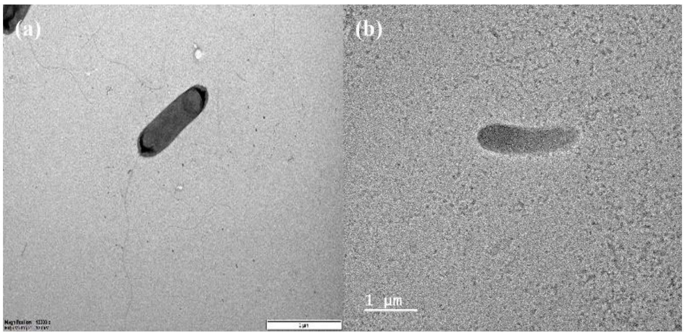

Fig. S3. Two-dimensional thin-layer chromatograms showing the polar lipid profiles of strains KJ10-1^T and KJ40-1^T. Solvent systems: (I) chloroform-methanol-water (65:25:4, v/v/v); (II) chloroform-acetic acid-methanol-water (80:15:12:4, v/v/v/v). The TLC plates were sprayed with 10% ethanolic molybdatophosphoric acid (a), ninhydrin (b), Dittmer-Lester (c), and *α*-naphthol (d) for the detection of total polar lipids, aminolipids, phospholipids, and glycolipids, respectively. Abbreviations: PE, phosphatidylethanolamine; PG, phosphatidylglycerol; DPG, diphosphatidylglycerol; AL, unidentified aminolipid.

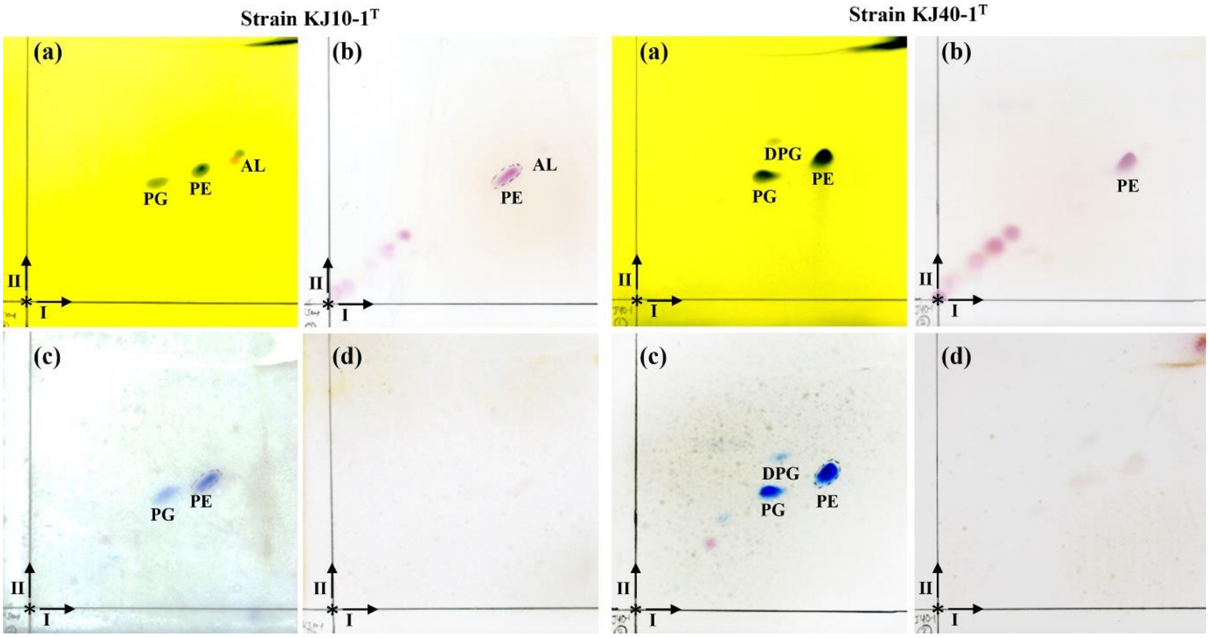

Table S1. The genomic relatedness between strain KJ10-1^T and closely related type strains of the genus *Shewanella*, as well as between strain KJ40-1^T and closely related type strains of the genus *Vibrio*

Taxa: 1, strain KJ10-1^T (JAODOQ000000000); 2, *S. saliphila* JCM 32304^T (BMQV00000000); 3, *S. algicola* JCM 31091^T (BMQI00000000); 4, *S. inventionis* CGMCC 1.15339^T (BMII00000000); 5, strain KJ40-1^T (JAQLOI000000000); 6, *V. hannami* KACC 19277^T (JARQZP000000000); 7, *V. rumoiensis* FERM P-14531^T (AP018685); 8, *V. marisflavi* WH134^T (CAKLDM000000000).

		**dDDH† value (%)**							
		**1**	**2**	**3**	**4**	**5**	**6**	**7**	**8**
**OrthoANI† value (%)**	**1**	–	47.4	43.4	28.8	–	–	–	–
	**2**	92.4	–	57.5	26.6	–	–	–	–
	**3**	91.2	94.5	–	26.7	–	–	–	–
	**4**	84.8	83.5	83.3	–	–	–	–	–
	**5**	–	–	–	–	–	20.8	24.1	23.1
	**6**	–	–	–	–	71.9	–	24.2	22.9
	**7**	–	–	–	–	70.9	70.7	–	23.8
	**8**	–	–	–	–	70.5	70.9	71.0	–

†dDDH, digital DNA-DNA hybridization; orthoANI, orthologous average nucleotide identity.

Table S2. Cellular fatty acid compositions (%) of strain KJ10-1^{T} and closely related type strains of the genus *Shewanella*

Taxa: 1, strain KJ10-1^{T}; 2, *S. saliphila* JCM 32304^{T}; 3, *S. algicola* JCM 31091^{T}; 4, *S. inventionis* KCTC 42807^{T}. All data were obtained from this study. Data are expressed as percentages of the total fatty acids, and fatty acids amounting less than 1.0% in all strains are not shown. Major components (>10.0%) are highlighted in bold; tr, trace amount (<1.0%); –, not detected.

Fatty acid	1	2	3	4
Saturated:				
$C_{12:0}$	1.1	4.7	2.0	3.5
$C_{13:0}$	tr	6.3	tr	1.5
$C_{14:0}$	2.3	**13.3**	3.1	2.5
$C_{16:0}$	**11.8**	tr	**12.5**	**11.4**
$C_{17:0}$	3.4	tr	1.3	2.3
Unsaturated:				
$C_{15:1}$ $\omega 8c$	2.1	6.8	1.2	2.1
$C_{15:1}$ $\omega 6c$	1.9	**11.1**	tr	1.5
$C_{17:1}$ $\omega 8c$	**13.7**	tr	6.1	**12.4**
$C_{17:1}$ $\omega 6c$	1.4	tr	–	tr
Branched:				
iso-$C_{13:0}$	4.0	6.3	7.5	2.4
iso-$C_{14:0}$	2.1	2.8	1.4	–
iso-$C_{15:0}$	**11.1**	**17.8**	**12.1**	5.6
Hydroxy:				
iso-$C_{12:0}$ 3-OH	tr	2.9	–	tr
$C_{12:0}$ 3-OH	tr	2.5	2.1	2.0
$C_{11:0}$ 3-OH	tr	1.5	tr	1.0
Summed feature*:				
1	tr	2.6	tr	tr
2	tr	1.4	1.0	1.1
3	**34.0**	5.9	**34.3**	**34.1**
8	3.5	tr	2.4	5.5

*Summed features are fatty acids that cannot be resolved reliably from another fatty acid using the chromatographic conditions chosen. The MIDI system groups these fatty acids together as one feature with a single percentage of the total. Summed features 1, 2, 3, and 8 comprise iso-$C_{15:1}$ H and/or $C_{13:0}$ 3-OH, iso-$C_{16:1}$ I and/or $C_{14:0}$ 3-OH, $C_{16:1}$ $\omega 7c$ and/or $C_{16:1}$ $\omega 6c$, and $C_{18:1}$ $\omega 7c$ and/or $C_{18:1}$ $\omega 6c$, respectively.

Table S3. Cellular fatty acid compositions (%) of strain KJ40-1^T and closely related type strains of the genus *Vibrio*

Taxa: 1, strain KJ40-1^T; 2, *V. hannami* KACC 19277^T; 3, *V. rumoiensis* DSM 19141^T; 4, *V. marisflavi* DSM 23086^T. All data were obtained from this study. Data are expressed as percentages of the total fatty acids, and fatty acids amounting less than 1.0% in all strains are not shown. Major components (>10.0%) are highlighted in bold; tr, trace amount (<1.0%); –, not detected.

**Fatty acid**	**1**	**2**	**3**	**4**
Saturated:				
$C_{12:0}$	4.4	4.4	4.4	2.6
$C_{14:0}$	6.1	5.1	2.3	5.1
$C_{16:0}$	**22.6**	**12.1**	**18.1**	**30.5**
$C_{18:0}$	1.2	1.7	5.1	4.9
Unsaturated:				
$C_{18:1}$ *ω9c*	–	–	1.1	–
$C_{18:1}$ *ω7c*	–	–	–	**14.5**
Branched:				
iso-$C_{15:0}$	–	tr	tr	1.2
iso-$C_{16:0}$	tr	tr	2.1	–
iso-$C_{17:0}$	tr	tr	–	1.1
Hydroxy:				
$C_{12:0}$ 3-OH	3.3	3.5	tr	2.9
Summed feature*:				
2	3.0	3.0	8.0	1.2
3	**39.4**	**37.4**	**41.9**	**30.3**
8	**13.7**	**28.6**	**15.6**	–

*Summed features are fatty acids that cannot be resolved reliably from another fatty acid using the chromatographic conditions chosen. The MIDI system groups these fatty acids together as one feature with a single percentage of the total. Summed features 2, 3, and 8 comprise iso-$C_{16:1}$ I and/or $C_{14:0}$ 3-OH, iso-$C_{15:0}$ 2-OH and/or $C_{16:1}$ *ω7c*/*ω6c*, and $C_{18:1}$ *ω7c* and/or $C_{18:1}$ *ω6c*, respectively.
